# Evaluation of Syndromic Surveillance Data for Studying Harmful Algal Bloom-Associated Illnesses — United States, 2017–2019

**DOI:** 10.15585/mmwr.mm7035a2

**Published:** 2021-09-03

**Authors:** Amy M. Lavery, Lorraine C. Backer, Virginia A. Roberts, Jourdan DeVies, Johnni Daniel, DHSc

**Affiliations:** ^1^Division of Environmental Health Science and Practice, National Center for Environmental Health, CDC; ^2^Division of Foodborne, Waterborne, and Environmental Diseases, National Center for Emerging and Zoonotic Infectious Diseases, CDC; ^3^Division of Health Informatics and Surveillance, Center for Surveillance, Epidemiology, and Laboratory Services, Centers for Disease Control and Prevention.

Harmful algal and cyanobacterial blooms (harmful algal blooms) are large colonies of algae or cyanobacteria that can harm humans, animals, and the environment (*1**–3*). The number of algal blooms has been increasing in the United States, augmented by increasing water temperatures and nutrients in water from industry and agricultural run-off (*4,5*). The extent to which harmful algal bloom exposures cause human illness or long-term health effects is unknown. As the number of blooms increases annually, the likelihood of negative health outcomes (e.g., respiratory or gastrointestinal illness) from exposure also increases (*4**,**5*). To explore the utility of syndromic surveillance data for studying health effects from harmful algal bloom exposures, CDC queried emergency department (ED) visit data from the National Syndromic Surveillance Program (NSSP) for harmful algal bloom exposure–associated administrative discharge diagnosis codes and chief complaint text terms related to harmful algal bloom exposure (*6*). A total of 321 harmful algal bloom-associated ED visits were identified during January 1, 2017–December 31, 2019. An increase in harmful algal bloom–associated ED visits occurred during warmer months (June–October), consistent with seasonal fluctuations of blooms and recent publications (*6,**7*). Although syndromic surveillance data are helpful for understanding harmful algal bloom–associated ED visits in the United States, exposures were documented infrequently with discharge diagnosis codes; 67% of harmful algal bloom–associated ED visits were identified through querying chief complaint text. Improving the documentation of harmful algal bloom exposures in medical records would further benefit future health studies.

NSSP is a collaboration among CDC, state, and local health departments, and academic and private sector partners which captures data electronically from EDs throughout the country. As of the end of the study period (December 2019), the national database represented approximately 70% of all ED visits in the United States. Data are queried by creating Boolean search terms of diagnostic codes and chief complaint text. Chief complaint text terms are also used to categorize visits into many broad, medically similar syndromes using prebuilt algorithms.

For the current analysis, a query was created that comprises main terms from the chief complaint (e.g., red tide, algae) along with discharge diagnostic codes associated with exposure to harmful algal blooms (*International Classification of Diseases, Tenth Revision, Clinical Modification* [ICD-CM-10]) codes and their corresponding Systematized Nomenclature of Medicine [SNOMED]* Clinical Terms codes). The final query was reviewed using the NSSP query development tool.^†^ Records identified by this query are defined as harmful algal bloom-associated ED visits. To exclude ED visits associated with the ingestion of contaminated seafood, relevant keywords such as “shellfish” or “ciguatera poisoning” and corresponding ICD-CM-10 codes (e.g., ciguatera poisoning, ICD-CM-10 code T61.0), were omitted from the query. Basic demographic information for patients with harmful algal bloom–associated ED visits was summarized by frequency and percentage. The number of identified harmful algal bloom–associated ED visits during 2017–2019 was described by U.S. Department of Health and Human Services region and visualized using a time series graph. Because the number of facilities reporting to NSSP has increased since 2017, regional and time series comparisons were shown as a percentage of total ED visits within NSSP. The frequencies with which various syndrome categories^§^ were recorded during the harmful algal bloom-associated ED visits were examined. Variables were created to indicate whether an ED visit was related to neurologic, gastrointestinal, respiratory, or dermatologic conditions.^¶ ^This activity was reviewed by CDC and was conducted consistent with applicable federal law and CDC policy.**

A total of 321 harmful algal bloom–associated ED visits were identified during January 1, 2017–December 31, 2019. Among these visits, 106 (33%) were identified through ICD-CM-10 codes only; the addition of chief complaint text key terms to the query identified an additional 215 visits. Harmful algal bloom–associated ED visits increased in the summer months (June–October) in all 3 years (Figure). A notable peak occurred in October 2018, corresponding with a large-scale red tide event in the Gulf of Mexico during August–November 2018; of the 197 ED visits occurring during July-November 2018, 73% occurred in Region 4 (southeastern United States).

**FIGURE:**
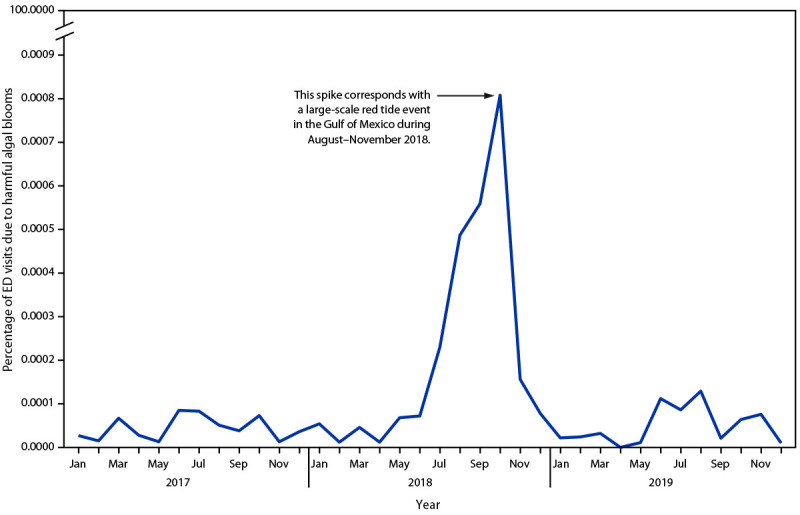
Harmful algal bloom exposure–associated emergency department visits among all emergency department visits, by month — National Syndromic Surveillance Program, United States, 2017–2019* * Percentage of all emergency department visits in the National Syndromic Surveillance Program was utilized to account for the increasing number of facilities contributing data to the National Syndromic Surveillance Program.

Harmful algal bloom–associated ED visits occurred primarily among patients aged 18–44 years (37%) and 45–64 years (30%) (Table 1); the majority (59%) occurred among females. The largest number of harmful algal bloom–associated ED visits was identified in Region 4 (31.1%). The most frequent syndrome category was respiratory (41%), followed by gastrointestinal (14%), neurologic (10%), and dermatologic (8%) (Table 2).

**TABLE 1:** Demographic characteristics of patients with harmful algal bloom-associated emergency department visits (n = 321) — National Syndromic Surveillance Program, United States, 2017–2019

Characteristic	No. (%)
**Age group (yrs)**	
0–4	19 (5.9)
5–17	35 (10.9)
18–44	118 (36.8)
45–64	96 (29.9)
≥65	50 (15.6)
Unknown	3 (0.9)
**Sex**	
Female	190 (59.2)
Male	131 (40.8)
HHS Region*^,†^	
1	13 (9.7)
2	13 (4.9)
3	11 (4.1)
4	213 (31.1)
5	29 (6.6)
6	8 (4.1)
7	5 (5.3)
8	7 (12.9)
9	11 (7.6)
10	11 (13.7)

**Abbreviation:** HHS = U.S. Department of Health and Human Services.

* https://www.hhs.gov/about/agencies/iea/regional-offices/index.html

^†^ Percentages for HHS regions are adjusted for the total number of emergency department visits during the time periods to account for the increasing number of facilities reporting to NSSP since 2017.

**TABLE 2:** Primary syndrome categories associated with harmful algal bloom exposure used among 321 harmful algal bloom-associated emergency department visits

Syndrome type	No. (%)*
Respiratory^†^	133 (41.4)
Gastrointestinal^§^	44 (13.7)
Neurologic^¶^	33 (10.3)
Dermatologic**	27 (8.4)

* Records could contain multiple syndromes. Percentages might not sum to 100% because of missing values or listings of other syndrome types that were not included for this analysis.

^†^ Respiratory symptoms consist of acute bronchitis, chest congestion, cough, difficulty breathing, sore throat, influenza-like illness, nasal congestion, otitis media, shortness of breath, upper respiratory infection, or wheezing.

^§^ Gastrointestinal symptoms consist of abdominal pain, diarrhea, gastrointestinal bleeding, loss of appetite, nausea, or vomiting.

^¶ ^Neurologic symptoms consist of altered mental status, dizziness, drowsiness, headache, or muscle weakness.

** Dermatologic symptoms consist only of rash.

## Discussion

This analysis identified approximately 300 harmful algal bloom–associated ED visits during 2017–2019. ED visits increased during the warmer months, consistent with seasonal patterns of harmful algal blooms in the environment, with a notable peak in 2018. Syndrome categories recorded for ED visits were consistent with harmful algal bloom exposures through inhalation (e.g., respiratory and neurologic), ingestion (e.g., gastrointestinal), or skin contact (e.g., dermatologic) (*5)*.

Most ED visits were identified through the chief complaint text rather than through the use of ICD-10-CM codes. These results corroborate an earlier analysis using a commercial claims data set, which identified few records with harmful algal bloom exposure ICD-10-CM codes (*8*). Searching the chief complaint text in NSSP more than doubled the number of harmful algal bloom–associated visits, compared with the number that would have been identified by searching on ICD-10-CM codes only. The peak in ED visits during 2018 occurred primarily within Region 4, corresponding to a large-scale red tide event in the Gulf of Mexico that persisted during June 2018–November 2018 (*9*). The occurrence of this peak at the time of a red tide event might explain the higher frequency of chief complaints associated with respiratory symptoms because red tide has been linked to respiratory health outcomes (*2,3*). Presumably, these types of large-scale events might cause providers to ask patients about recent harmful algal bloom exposures or cause patients to mention them.

The NSSP query development tool made it possible to review a sample of the full chief complaint text without linking to other visit data, which helped to protect patient anonymity. Several chief complaints (six) used terms such as, “patient denies red tide exposure.” The final query was adjusted to exclude these records; however, this finding implies that providers might have been asking patients if they had been exposed to red tide, or patients might have mentioned that they had not been on the beach or exposed to red tide before their ED visit. Increasing awareness so that more patients know to mention harmful algal bloom exposure and more physicians know to ask about harmful algal blooms would enhance understanding of harmful algal bloom–associated ED visits. 

The findings in this report are subject to at least two limitations. First, some records might have been misclassified or miscoded. For example, the query development tool identified some records with a chief complaint that seemed unrelated to harmful algal bloom exposure (e.g., meningitis exposure or vaginal problems) despite the use of the Z77.121 harmful algal bloom exposure ICD-10-CM code. In addition, it is unknown what occurred during the ED visit between when the chief complaint was assigned at triage and when the final diagnosis was determined. Some patients might have described a harmful algal bloom exposure, but medical personnel might have ruled it out as the primary reason for diagnosis. Second, NSSP undercounts the number of harmful algal bloom–associated ED visits that resulted from environmental exposures because 1) only 70% of ED visits nationally are included within the data set, and 2) ICD-10-CM codes are from billing data and codes for harmful algal bloom exposures might not be included if they do not affect reimbursement. Despite these limitations, however, these analyses provide information of how often exposure to a harmful algal bloom is documented during ED visits through diagnostic codes and chief complaints.

These findings provide information about how harmful algal bloom exposure can be identified through syndromic surveillance ED visit data and potentially used to identify the extent of illness from harmful algal bloom exposure in the United States. As the frequency and geographic extent of harmful algal blooms increase, it is important for health care providers to discuss and document harmful algal bloom exposures and health effects during medical visits to ensure proper patient treatment and help patients understand how to prevent exposure in the future. As access to information from electronic medical records for research improves, better documentation of harmful bloom exposures and illnesses can help support a more accurate assessment of their acute public health impact. With better documentation, electronic health record systems with longitudinal data could potentially provide data for monitoring long-term health effects from these exposures, the extent of which are largely unknown.

**Summary**

**What is already known about this topic?**

Harmful algal and cyanobacterial blooms are large colonies of algae or cyanobacteria that can harm humans, animals, and the environment.

**What is added by this report?**

National syndromic surveillance data identified 321 emergency department visits related to harmful algal bloom exposure during 2017–2019. Frequency of these visits was highest during warmer months.

**What are the implications for public health practice?**

Syndromic surveillance data are useful for studying the extent of harmful algal bloom–associated illness. Increasing awareness so that more patients know to mention harmful algal bloom exposures and more physicians know to ask about them could improve documentation of health effects and enable further use of health records for health studies.

## Footnotes

† The NSSP Chief Complaint Query Validation data source contains chief complaint and discharge diagnosis codes only; to help protect anonymity, it does not include any demographic data. This data subset helps users to iteratively develop queries with inclusion and exclusion terms to capture only the records of interest. Some NSSP sites do not contribute data to this data source. The final query included the following ICD-10-CM codes: T65.82, toxic effect harmful algae and algae toxins; Z77.121, contact with and suspected exposure to harmful algae and algae toxins; SNOMED codes: 137512, 240914003, 10076437, 10076441, 402161005, 702986006, and 81034007; and main terms related to algae and red tide. *International Classification of Diseases, Ninth Edition* (ICD-9) codes were not included because ICD-10-CM codes for HAB exposure were implemented in October 2015 and were similar, but more descriptive than the ICD-9 codes for HAB exposure. Other terms related to HAB exposure, such as “cyanobacteria” and “hab,” did not identify additional records and were not included in the final query. The final query did not include ICD-10-CM codes for HAB exposures through seafood or shellfish poisonings.
